# Molecular dynamic analysis of the functional role of amino acid residues V99, F124 and S125 of human DNA dioxygenase ABH2

**DOI:** 10.18699/vjgb-25-111

**Published:** 2025-12

**Authors:** M. Zhao, T.E. Tyugashev, A.T. Davletgildeeva, N.A. Kuznetsov

**Affiliations:** Novosibirsk State University, Novosibirsk, Russia; Institute of Chemical Biology and Fundamental Medicine of the Siberian Branch of the Russian Academy of Sciences, Novosibirsk, Russia; Institute of Chemical Biology and Fundamental Medicine of the Siberian Branch of the Russian Academy of Sciences, Novosibirsk, Russia; Novosibirsk State University, Novosibirsk, Russia Institute of Chemical Biology and Fundamental Medicine of the Siberian Branch of the Russian Academy of Sciences, Novosibirsk, Russia

**Keywords:** DNA repair, base methylation, human DNA dioxygenase ABH2, MD modeling, functional role of amino acid residues, репарация ДНК, метилирование оснований, ДНК-диоксигеназа человека ABH2, MД-моделирование, функциональная роль аминокислотных остатков

## Abstract

The ABH2 enzyme belongs to the AlkB-like family of Fe(II)/α-ketoglutarate-dependent dioxygenases. Various non-heme dioxygenases act on a wide range of substrates and have a complex catalytic mechanism involving α-ketoglutarate and an Fe(II) ion as a cofactor. Representatives of the AlkB family catalyze the direct oxidation of alkyl substituents in the nitrogenous bases of DNA and RNA, providing protection against the mutagenic effects of endogenous and exogenous alkylating agents, and also participate in the regulation of the methylation level of some RNAs. DNA dioxygenase ABH2, localized predominantly in the cell nucleus, is specific for double-stranded DNA substrates and, unlike most other human AlkB-like enzymes, has a fairly broad spectrum of substrate specificity, oxidizing alkyl groups of such modified nitrogenous bases as, for example, N 1-methyladenosine, N 3-methylcytidine, 1,N 6-ethenoadenosine and 3,N 4-ethenocytidine. To analyze the mechanism underlying the enzyme’s substrate specificity and to clarify the functional role of key active-site amino acid residues, we performed molecular dynamics simulations of complexes of the wild-type ABH2 enzyme and its mutant forms containing amino acid substitutions V99A, F124A and S125A with two types of DNA substrates carrying methylated bases N 1-methyladenine and N 3-methylcytosine, respectively. It was found that the V99A substitution leads to an increase in the mobility of protein loops L1 and L2 involved in binding the DNA substrate and changes the distribution of π-π contacts between the side chain of residue F102 and nitrogenous bases located near the damaged nucleotide. The F124A substitution leads to the loss of π-π stacking with the damaged base, which in turn destabilizes the architecture of the active site, disrupts the interaction with the iron ion and prevents optimal catalytic positioning of α-ketoglutarate in the active site. The S125A substitution leads to the loss of direct interaction of the L2 loop with the 5’-phosphate group of the damaged nucleotide, weakening the binding of the enzyme to the DNA substrate. Thus, the obtained data revealed the functional role of three amino acid residues of the active site and contributed to the understanding of the structural-functional relationships in the recognition of a damaged nucleotide and the formation of a catalytic complex by the human ABH2 enzyme.

## Abstract (ru)

ДНК-диоксигеназа человека ABH2 относится к семейству AlkB-подобных негемовых диоксигеназ, которые действуют на широкий спектр субстратов и обладают сложным каталитическим механизмом с участием α-кетоглутарата и иона Fe(II) в качестве кофактора. Представители семейства AlkB катализируют прямое окисление алкильных заместителей в азотистых основаниях ДНК и РНК, обеспечивая защиту от мутагенного воздействия эндогенных и экзогенных алкилирующих агентов, а также участвуя в регуляции уровня метилирования некоторых РНК. Фермент ABH2, локализованный преимущественно в ядре клетки, проявляет специфичность к двуцепочечным ДНК-субстратам и, в отличие от большинства других AlkB-подобных ферментов человека, обладает довольно широким спектром субстратной специфичности, окисляя алкильные группы таких модифицированных азотистых оснований, как, например, N 1-метиладенозин, N 3-метилцитидин, 1,N 6-этеноаденозин и 3,N 4-этеноцитидин. В данной работе с целью анализа механизма, обеспечивающего субстратную специфичность фермента, и выяснения функциональной роли аминокислотных остатков в составе активного центра нами выполнено молекулярно-динамическое моделирование комплексов фермента ABH2 дикого типа и его мутантных форм, содержащих аминокислотные замены V99A, F124A или S125A, с двумя типами ДНК-субстратов, несущих метилированные основания N 1-метиладенин или N 3-метилцитозин. Установлено, что замена V99A приводит к увеличению подвижности белковых петель L1 и L2, участвующих в связывании ДНК-субстрата, и изменяет распределение π-π-контактов боковой цепи остатка F102 с азотистыми основаниями, расположенными рядом с поврежденным нуклеотидом. Замена F124A приводит к потере π-π-стэкинга с поврежденным основанием, что, в свою очередь, дестабилизирует архитектуру активного центра, вызывает нарушение взаимодействия с ионом железа и препятствует оптимальному каталитическому позиционированию α-кетоглутарата в активном центре. Замена S125A приводит к потере прямого взаимодействия петли L2 с 5’-фосфатной группой поврежденного нуклеотида, ослабляя связывание фермента с ДНК-субстратом. Таким образом, полученные данные позволили установить функциональную роль трех аминокислотных остатков ак- тивного центра и расширить понимание структурно-функциональных связей в процессах узнавания повреж- денного нуклеотида и формирования каталитического комплекса ферментом ABH2 человека.

## Introduction

The stability of genetic information encoded in the form of nucleotide sequences in DNA is extremely important for normal functioning and survival of individual cells, organisms, and species as a whole (Travers, Muskhelishvili, 2015). At the same time, cellular DNA of all living organisms is regularly subjected to damaging effects of various endogenous and exogenous factors, such as chemically reactive reagents and metabolites, ionizing and UV radiation, and others (Ougland et al., 2015). Living organisms evolved multiple different repair pathways for damage occurring in genomic DNA, some of which are represented by a single enzyme, while others involve sequential and coordinated work of entire enzymatic cascades (Yi et al., 2009; Li et al., 2013; Müller, Hausinger, 2015; Ougland et al., 2015).

So, among enzymes participating in recognition and removal of non-bulky individual damage to DNA nitrogenous bases, the following can be distinguished: 1) DNA glycosylases that remove damaged nitrogenous bases with the formation of apurinic/apyrimidinic sites in DNA, which are then processed with restoration of the original DNA structure by other enzymes of the base excision repair (BER) pathway (Ringvoll et al., 2006; Chen et al., 2010; Li et al., 2013); 2) О6-alkylguanine-DNA-alkyltransferases (AGT) that transfer the alkyl adduct to their own cysteine residue (Ringvoll et al., 2006); 3) photolyases responsible for the removal of UV-induced photodamage such as cyclobutane pyrimidine dimers and pyrimidine-pyrimidine photoproducts (Yi, He, 2013); 4) dioxygenases of the AlkB family, belonging to the superfamily of Fe(II)/α-ketoglutarate(αKG)-dependent dioxygenases that use non-heme iron as a cofactor and αKG as a cosubstrate for direct oxidation of alkyl groups in damaged DNA bases (Yang et al., 2009; Yi et al., 2009; Kuznetsov et al., 2021). It should be noted that the diversity of repair pathways for non-bulky DNA lesions is related to the great diversity of possible chemical modifications of nitrogenous bases.

Representatives of the Fe(II)/αKG- dependent dioxygenase AlkB family found in humans have attracted particular interest in recent years due to their participation in the repair of alkylated DNA bases. It is believed that enzymes of this family may play an important role in the progression of some oncological diseases since they are often overexpressed in tumor cells and neutralize the effect of alkylating drugs used in chemotherapy. ABH2 is one of the first identified human representatives of the AlkB-like dioxygenase family (Duncan et al., 2002; Aas et al., 2003). It is known that changes in ABH2 expression levels affect the efficiency of removal of certain toxic DNA damages in tumor cells, making this enzyme a potential marker for cancer diagnostics and a possible therapeutic target (Wilson et al., 2018).

**Fig. 1.**
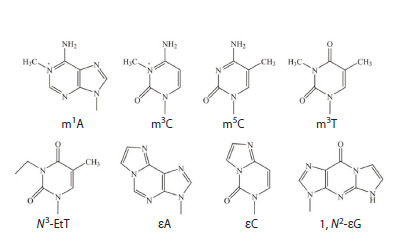
Alkylated nitrogenous bases that are substrates for human DNA dioxygenase ABH2

To date, it is known that ABH2 exhibits activity against at least 8 different alkylated DNA bases, namely N 1-methyladenosine (m1A), N 3-methylcytidine (m3C), N 3-methylthymidine (m3T), N 3-ethylthymidine (N3-EtT), 1,N 6-ethenoadenosine (εA), 3,N 4-ethenocytidine (εC), 1,N 2-ethenoguanosine (1,N 2-εG), and 5-methylcytidine (m5C) (Fig. 1) (Falnes, 2004; Ringvoll et al., 2006, 2008; Bian et al., 2019).

Methylation is the most common type of DNA base damage caused by exposure to alkylating agents (Sall et al., 2022), and m1A and m3C are substrates most effectively removed by ABH2 from double-stranded DNA (dsDNA) (Duncan et al., 2002; Aas et al., 2003; Xu et al., 2021). D.H. Lee et al. showed that ABH2 oxidizes m1A and m3C in the context of dsDNA at least twice as efficiently compared to single-stranded DNA (ssDNA) (Lee et al., 2005).

Currently known structural data allow suggestion of specific features of ABH2 enzyme functioning and the mechanism providing its substrate specificity. ABH2 contains a highly conserved catalytic domain – a double-stranded β-helical domain (DSBH) of the Fe(II)/αKG-dependent dioxygenase superfamily. The unstructured N-terminal fragment of ABH2 also includes a proliferating cell nuclear antigen (PCNA) binding motif (Xu et al., 2021). A triplet consisting of two histidine amino acid residues and one aspartate (H171, H236, and D173) coordinates the Fe(II) cofactor in the enzyme’s active site (Giri et al., 2011; Xu et al., 2021). D173 amino acid residue, through interaction with R254 residue, also participates in formation of a hydrogen bond network including N159, Y161, R248, T252, and R254 amino acid residues, that coordinate the αKG cosubstrate in the enzyme’s active site (Waheed et al., 2020).

**Fig. 2.**
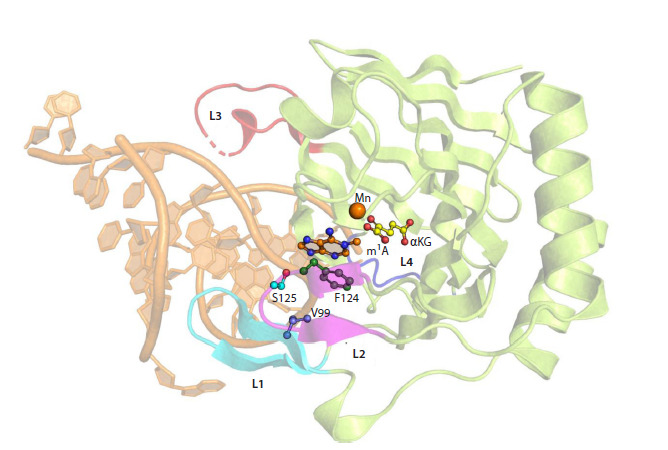
Crystal structure of the ABH2 complex with dsDNA containing m1A (PDB ID 3BUC). Loops L1–L4 are marked, damaged nitrogenous base m1A, αKG and Mn2+ ion, as well as the amino acid residues considered in this work (V99, F124, and S125) are shown.

The ABH2 active site is surrounded by four functional loops, L1-L4 (Fig. 2). These loops play a key role in stabilizing the position of the DNA substrate in the enzyme’s active site (Xu et al., 2021). Loop L1, including amino acid residues 98–107, contains a hydrophobic hairpin V101-F102-G103, through which “testing” of base pair stability in the substrate occurs. If a damaged base forms an unstable pair with its partner from the complementary strand, V101 and F102 residues induce flipping of the damaged nucleotide into the active site. Herewith the vacated space in the DNA duplex is filled by F102 residue, stabilizing the flipped-out position of the nucleotide through π-π interaction with the surrounding bases (Chen et al., 2010, 2014; Yi et al., 2012; Xu et al., 2021).

Loop L2, including amino acid residues 122–129, together with loop L1 forms the so-called “nucleotide recognition lid” (NRL). Y122 amino acid residue participates in a hydrogen bond network forming the catalytically competent state of the enzyme’s active site (Davletgildeeva et al., 2023), S125 residue forms a hydrogen bond with the 5′-phosphate of the flipped damaged nucleotide; F124 and H171 amino acid residues form π-π stacking with the flipped nitrogenous base (Chen et al., 2010, 2014; Lenz et al., 2020). S125 amino acid residue also participates in forming the wall of the damagebinding pocket alongside V99, R110, and I168 residues (Davletgildeeva et al., 2023).

It should be noted that V99 holds an important position in the network of hydrophobic residues formed by V101, V108, F124, and L127 (Monsen et al., 2010). Loop L3, including amino acid residues 198–213, and loop L4, including amino acid residues 237–247, play an important role in binding to the dsDNA substrate. R198, R203, and K205 amino acid residues in loop L3 and the RKK sequence (R241-K242-K243) in loop L4 form contacts with the DNA strand complementary to the damaged strand, thereby ensuring effective binding of the dsDNA substrate by the ABH2 enzyme (Yang et al., 2008; Yi et al., 2009; Waheed et al., 2020).

Molecular dynamic analysis of structural data and experimental verification of activity of recombinant preparations of wild-type ABH2 and several of its mutant forms, conducted by our group previously, allowed establishment of the role of Y122, I168, and D173 amino acid residues, which form direct contacts with bases m1A, m3C, as well as m5C, in the active site pocket (Davletgildeeva et al., 2023). Comparative analysis of enzymes revealed the influence of substitutions of these amino acid residues on the enzyme’s catalytic activity, and only a slight decrease in DNA binding efficiency. The obtained data suggested that these residues are responsible for precision positioning of the flipped damaged nucleotide in the active site, which ensures effective catalytic reaction (Davletgildeeva et al., 2023).

It should be noted that the broad spectrum of substrate specificity of the ABH2 enzyme and the complex catalytic mechanism of action, including cofactor and cosubstrate for reaction implementation, complicate detailed studies of the molecular mechanism of damaged DNA recognition and catalytically competent complex formation as well as local conformational changes affecting catalytic reaction efficiency. Due to the above, in the present study, with the aim of further elucidating the mechanism of substrate specificity of human DNA dioxygenase ABH2 using molecular dynamics methods, analysis of the functional role of three amino acid residues, V99, F124, and S125, participating in the formation of the pocket where the flipped-out damaged nucleotide is located, was conducted.

## Materials and methods

Complex models were built based on crystallographic structures of the ABH2-dsDNA complexes with metal ion (Mn2+) and αKG: 3BUC (for m1A), and 3RZJ (for m3C) (Yang et al., 2008; Yi et al., 2012). DNA sequence changes, correction of unresolved amino acid residues and enzyme modifications were performed using Chimera and Modeller (Šali, Blundell, 1993), protonation optimization of ionizable groups was done using the H++ server (Anandakrishnan et al., 2012). MD modeling was performed in GROMACS (Abraham et al., 2015). The complex was placed in a dodecahedral cell with TIP3P water and 50 mM KCl (Jorgensen et al., 1983; Joung, Cheatham, 2008), the AMBER14SB/OL15 force field was used to describe the complex (Cornell et al., 1996; Zgarbová et al., 2011, 2015; Maier et al., 2015).

Parameterization for m1A, m3С and αKG was performed using the Antechamber module (AMBER package), RESP charges were calculated on the REDD server, topologies of modified residues were converted to GROMACS format using ACPYPE (Bayly et al., 1993; Wang et al., 2004, 2006; Vanquelef et al., 2011; Sousa da Silva, Vranken, 2012).In order to preserve octahedral coordination geometry of Fe2+ ion under possible active site perturbations introduced by amino acid residue substitutions, a distributed charge model was used to describe the ion (Jiang et al., 2016). The following parameters were used for MD calculations: system energy minimization by the steepest descent method, van der Waals interaction cutoff value set to 10 Å, long-range Coulomb interactions accounted for by the PME (Particle Mesh Ewald) method (Essmann et al., 1995), hydrogen atom covalent bond vibration restriction by the LINCS method (Hess et al., 1997).

After minimization, the system was heated to 310 K in NVT ensemble for 500 ps using a V-rescale thermostat (Bussi et al., 2007). Then equilibration in NPT ensemble was performed for 1 ns, pressure was maintained at 1 bar using a Parrinello–Rahman barostat (Parrinello, Rahman, 1981).

Classical molecular dynamics calculations were performed for 250 ns duration at least three times. Trajectory analysis was performed using built-in GROMACS tools and the MDTraj library (McGibbon et al., 2015). Distribution changes between stable states of wild-type ABH2 enzyme complexes and its mutant forms with DNA substrates are shown in distance distribution graphs between key atoms during modeling. Interatomic distance distributions in MD trajectory are presented as histograms with 0.1 Å step and step height equal to the percentage of trajectory frames in which the distance falls within the corresponding range of values. For each trajectory, the sum of fractions across the entire distance range equals 100 %.

## Results

**Model of the ABH2 V99A enzyme-substrate complex with damaged DNA**

**Fig. 3.**
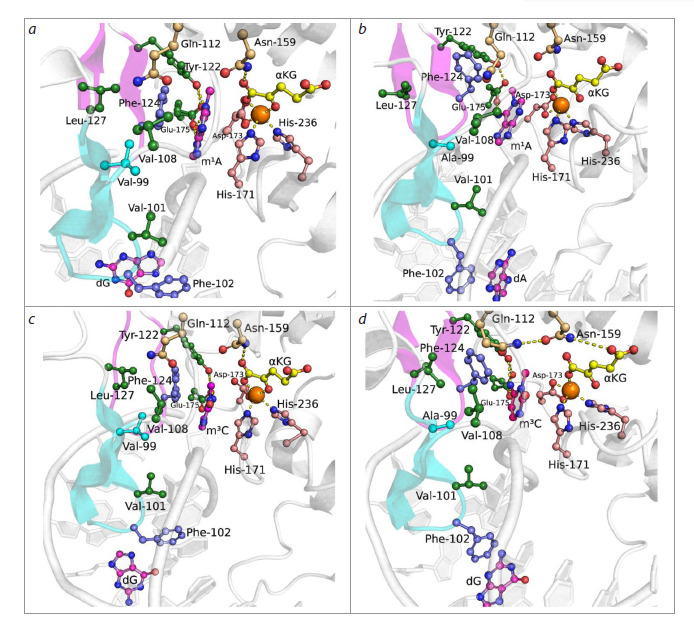
Representative MD structures of ABH2 WT in complex with m1A-DNA (a) and m3C-DNA (c), and ABH2 V99A in complex with m1A-DNA (b) and m3C-DNA (d). Key amino acid residues of the active site, damaged nitrogenous base, αKG and Mn2+ ion are shown. Loops L1 (blue) and L2 (pink) are highlighted with corresponding colors.

**Fig. 4.**
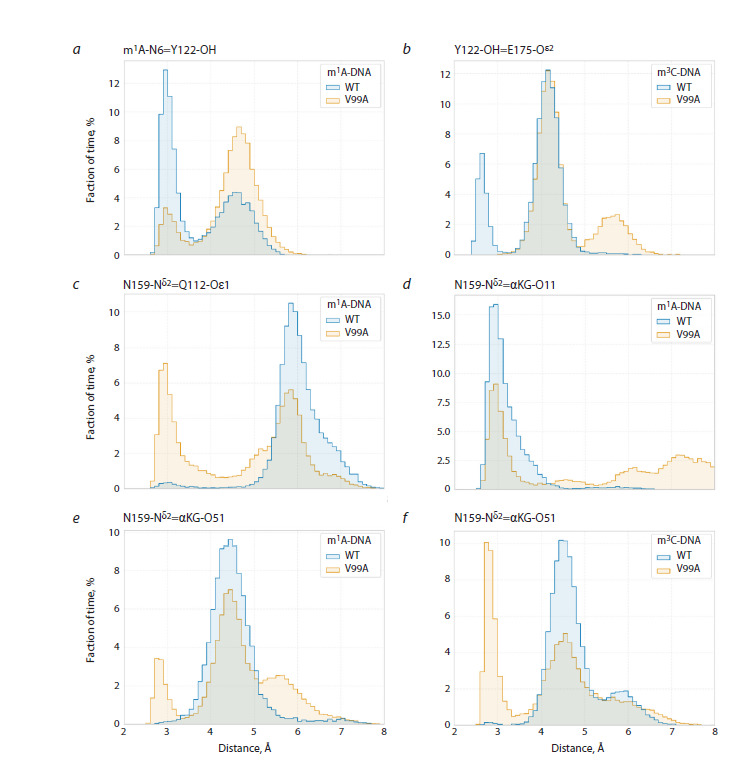
Distance distributions between key atoms when modeling complexes of the wild-type ABH2 enzyme and its V99A mutant form with DNA substrates

When modeling enzyme-substrate complexes both with the m1A-containing dsDNA substrate (hereafter m1A-DNA, Fig. 3a, b), and with the m3C-containing dsDNA substrate (hereafter m3C-DNA, Fig. 3c, d), the V99A substitution led to changes both in the region of loops L1 and L2 interacting with the nucleotide flipped into the enzyme’s active site and the adjacent dsDNA region, and in the cosubstrate binding region. Thus, in the model complex with m1A-DNA, the side chain of F124 amino acid residue lost π-π stacking interactions with the base of the nucleotide flipped into the active site (Fig. 3a, b). This reduced the lifetime of the hydrogen bond between the hydroxyl group of Y122 and the exocyclic amino group of the damaged base (Fig. 4a). In the model complex with the m3C-containing dsDNA substrate, partial loss of contact between the hydroxyl group of the Y122 side chain and the carboxyl group of the E175 side chain also occurred (Fig. 4b), which also disrupted the contact network stabilizing the flipped-out base.

The V99A substitution induced a change in the position of F102 residue, which intercalates into DNA and is part of loop L1. Herewith, in the complex with m1A-DNA, redistribution of π-π contacts formed by F102 occurred from the nitrogenous base of the complementary strand in the wild-type enzyme (dG in Fig. 3a) to the nitrogenous base of the damaged strand in case of ABH2 V99A (dA in Fig. 3b).

The values of the dihedral angle C-Cα-Cβ-Cγ at F102 residue were 148.1 ± 55.3° for wild-type enzyme and 127.2 ± 47.7° for the V99A mutant form, indicating stability of these positions during molecular dynamics. Meanwhile, in the complex with m3C-DNA, the V99A substitution induced a significant increase in the mobility of its side chain (dihedral angle C-Cα-Cβ-Cγ equals 135.6±58.6° and 100.2±100.3° for the wild-type enzyme and V99A, respectively). Increased mobility of F102 residue led to guanine complementary to m3C (dG in Fig. 3c, d) acquiring the opportunity to return inside the DNA structure in the mutant enzyme complex, entering into π-π contact with the side chain of F102, while this guanine was completely flipped out from the DNA double strand in the wild-type enzyme complex

The V99A substitution also induced changes in interaction with the cosubstrate, which led to αKG adopting a catalytically unfavorable conformation for half of the total modeling trajectory time. Changes in position of hydrophobic residues V108, F124, L127, and L129 in loops L1 and L2 lead to reorientation of amino acid residues Q112 and N159. In turn, in the wildtype enzyme, the side chain of N159 is one of the elements of the contact network supporting catalytically competent orientation of the cosubstrate, forming a hydrogen bond with the α-carboxyl group of αKG. Convergence of side chains of Q112 and N159 residues in the ABH2 V99A mutant form (Fig. 4c) leads to transfer of the hydrogen bond of the amide group of N159 from the α-carboxyl group of αKG (Fig. 4d) to the ω-carboxyl group of αKG (Fig. 4e, f), provoking its displacement from the optimal position for catalysis

Thus, modeling results allow the suggestion that the V99A substitution, leading to disruptions in the binding of both substrate and cosubstrate in the enzyme’s active site, should cause significant activity reduction. These data are in a good agreement with experimental results obtained previously for the V99A mutant form, revealing significant reduction (Monsen et al., 2010) or complete loss (Davletgildeeva et al., 2025) of ABH2 V99A catalytic activity toward dsDNA substrates containing m1A or m3C as damage

**Model of the ABH2 F124A enzyme-substrate complex with damaged DNA**

**Fig. 5.**
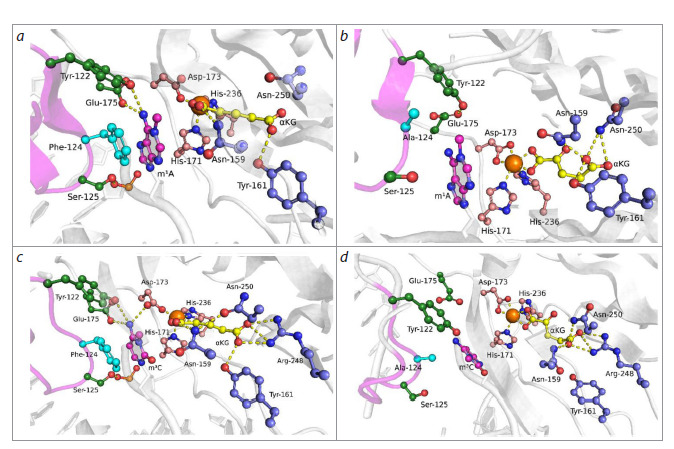
Representative MD structures of complexes ABH2 WT with m1A-DNA (a) and m3C-DNA (c), and ABH2 F124A with m1A-DNA (b) and with m3C-DNA (d). Key amino acid residues of the active site, damaged nitrogenous base, αKG and Mn2+ ion are shown. Loop L2 is highlighted with color (pink).

To determine the functional role of F124 residue, modeling of complexes of the ABH2 F124A mutant form with m1A- and m3C-containing dsDNA was performed (Fig. 5). Detailed analysis of distribution changes of distances between key atoms of the active site in case of F124A substitution revealed destabilization of both the flipped methylated nitrogenous base and αKG in the enzyme’s active site

**Fig. 6.**
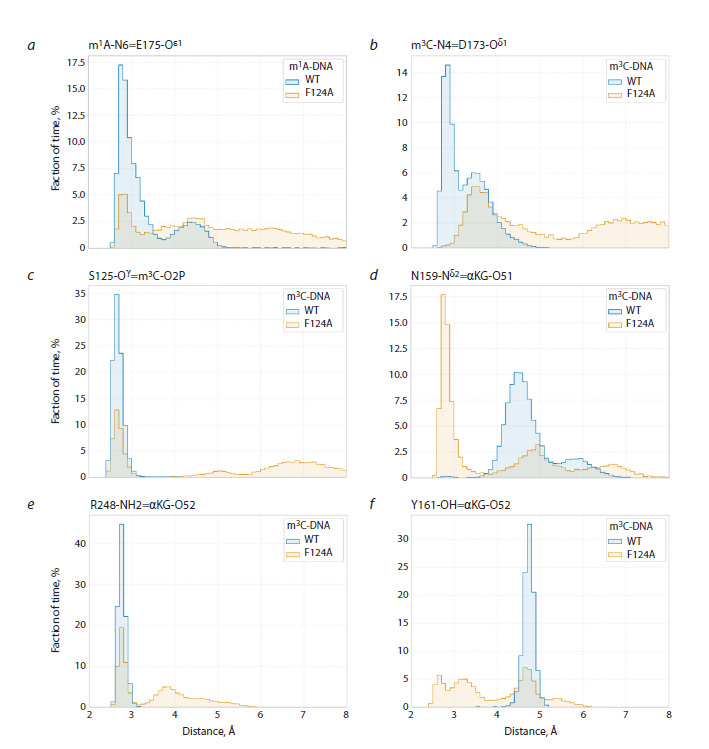
Distance distributions between key atoms when modeling complexes of the wild-type ABH2 enzyme and its F124A mutant form with DNA substrates

Thus, the F124A substitution, directly leading to loss of π-π stacking between the F124 side chain and the nitrogenous base, induces rotation and displacement of the flipped base from the enzyme’s active site, with concomitant loss of hydrogen bonds with side chains of Y122, D173, E175 residues (Fig. 6a, b). The hydrogen bond between the hydroxyl group of S125 residue and the corresponding phosphate group of the nucleotide backbone is also lost, reflecting deterioration of contact between loop L2 and DNA (Fig. 6c).The cosubstrate also loses catalytically competent position as a result of restructuring of the hydrogen bond network involving amino acid residues coordinating it. The amide group of N159 maintains a hydrogen bond predominantly with the ω-carboxyl group of αKG instead of the α-carboxyl group (Fig. 6d). Destabilization of the cosubstrate position is reflected in changes in the nature of contacts between side chains of Y161 and R248 residues and the ω-carboxyl group of αKG. If in the wild-type enzyme complex, stable hydrogen bonds are maintained between the guanidinium group of R248 and O2 atom of the ω-carboxyl group of αKG, and between the hydroxyl group of Y161 and O1 atom of the ω-carboxyl group, then in the ABH2 F124A mutant form complex, expansion of these distance distributions occurs, indicating contact destabilization (Fig. 6d, e).

The results of modeling indicate that amino acid residue F124 plays an important role in the structure of the ABH2 enzyme active site. This conclusion agrees with data (Chen et al., 2010; Monsen et al., 2010), as well as with data obtained previously in our laboratory (Davletgildeeva et al., 2025), according to which the ABH2 F124A mutant form completely lost catalytic activity toward m1A- and m3C-containing DNA substrates.

**Model of the ABH2 S125A enzyme-substrate complex with damaged DNA**

**Fig. 7.**
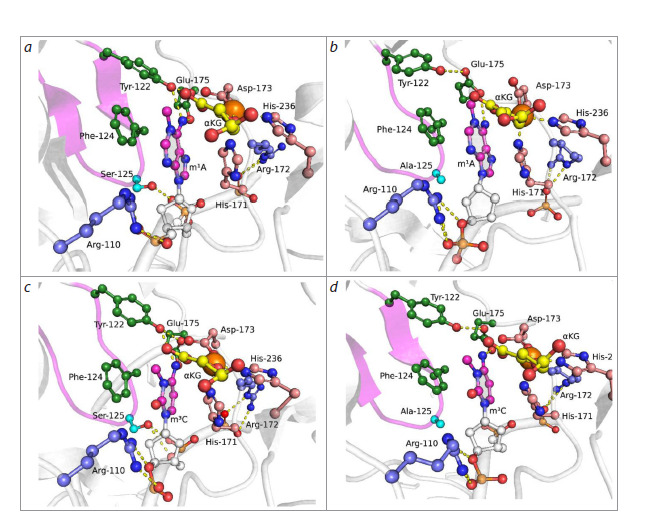
Representative MD structures of complexes ABH2 WT with m1A-DNA (a) and m3C-DNA (c), and ABH2 S125A with m1A-DNA (b) and with m3C-DNA (d). Key amino acid residues of the active site, damaged nitrogenous base, αKG and Mn2+ ion are shown. Loop L2 is highlighted with color (pink).

**Fig. 8.**
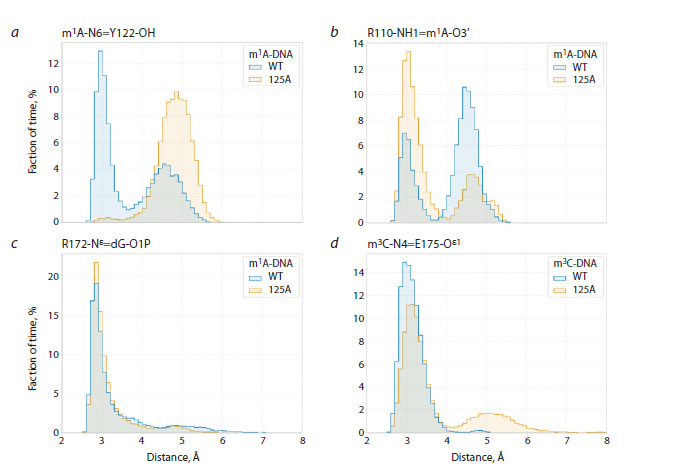
Distance distributions between key atoms when modeling complexes of the wild-type ABH2 enzyme and its S125A mutant form with DNA substrates.

The S125A substitution in the ABH2 enzyme causes loss of the hydrogen bond between the hydroxyl group of the amino acid residue and the 5′-phosphate group of the damaged nucleotide, leading to loss of direct interaction of loop L2 with m1A- (Fig. 7a, b) and m3C-DNA (Fig. 7c, d). Analysis of distance changes between key residues of the active site showed that in the enzyme complex with m1A-DNA, loss of loop L2 interaction with DNA causes loss of the hydrogen bond between the hydroxyl group of Y122 residue from L2 and the exocyclic amino group of m1A (Fig. 8a). At the same time, convergence of guanidinium groups of R110 and R172 residues with the O3′ atom of the nucleotide of the flipped nitrogenous base and the О5′ atom of the nucleotide located 5′ to the flipped nitrogenous base, respectively, occurs (Fig. 8b, c). Thus, in case of DNA substrate containing m1A, the S125A substitution leads to R110 and R172 amino acid residues binding more strongly to the DNA sugar-phosphate backbone

Unlike the ABH2 S125A enzyme complex with m1ADNA, in the model complex with m3C-DNA, convergence of guanidinium groups of R110 and R172 residues with the sugar-phosphate backbone does not occur (Fig. 7c, d). Meanwhile, compared to the WT enzyme, in case of S125A substitution, stability of the hydrogen bond between the side chain of E175 residue and the exocyclic amino group of m3С decreases (Fig. 8d).

Deterioration of direct contact with the flipped base and possible compensatory restructuring in case of S125A substitution in the ABH2 active site agrees with the results obtained by B. Chen et al., since their work showed that the ABH2 S125A mutant form retains catalytic activity toward dsDNA containing m1A as damage (Chen et al., 2010). However, in a later work (Davletgildeeva et al., 2025), it was shown that this substitution leads to loss of ABH2 catalytic activity toward both m3C- and m1A-containing DNA under the used reaction conditions. This suggests that compensatory restructuring that occurs according to modeling data in the ABH2 structure upon S125A substitution cannot fully preserve the enzyme’s catalytic activity on all types of DNA substrates

## Conclusion

Introduction of the V99A substitution into the ABH2 enzyme affected other amino acid residues forming the hydrophobic network of which the substituted residue is a part. This led to negative influence on functional loops L1 and L2, causing destabilization of their position, which, in turn, led to reorientation or displacement of key amino acid residues, Y122, E175, and F102, comprised in these loops. Additionally, the V99A substitution led to a catalytically unfavorable conformation of αKG in the enzyme’s active site. The obtained data confirm the role of V99 amino acid residue as an important participant in intraprotein coordination necessary for effective oxidation of methyl groups in damaged DNA bases by the ABH2 enzyme.

Substitution of amino acid residue F124, localized in NRL, led to significant displacement of both L1 and L2 loops and the damaged base itself relative to each other due to loss of π-π stacking with the damaged nitrogenous base. This substitution also led to changes in Fe2+ ion coordination, both through changes in coordination type by the αKG molecule and through additional coordination by D173 amino acid residue. The obtained data suggest extreme importance of F124 amino acid residue in the catalytic process carried out by ABH2 DNA dioxygenase.

The S125A substitution led to loss of direct interaction of loop L2 with the 5′-phosphate group of the damaged nucleotide; however, according to MD modeling data, this contact can be partially compensated by formation of bonds between R110 and R172 amino acid residues and the DNA sugar-phosphate backbone. It should be noted that such contact compensation was found only in case of the ABH2 S125A complex with m1A-containing DNA substrate, but not in case of m3C, which indirectly indicates a more complex mechanism responsible for recognition of different damages in the enzyme’s active site.

Thus, the MD modeling data obtained in the present work for complexes of human ABH2 DNA dioxygenase mutant forms containing V99A, F124A, or S125A amino acid substitutions with m1A- and m3C-containing DNA substrates indicate the important role of all three amino acid residues in ensuring formation of a catalytically competent state of the active site when interacting with damaged DNA.

## Conflict of interest

The authors declare no conflict of interest.
